# Phage selection restores antibiotic sensitivity in MDR *Pseudomonas aeruginosa*

**DOI:** 10.1038/srep26717

**Published:** 2016-05-26

**Authors:** Benjamin K. Chan, Mark Sistrom, John E. Wertz, Kaitlyn E. Kortright, Deepak Narayan, Paul E. Turner

**Affiliations:** 1 Department of Ecology and Evolutionary Biology, Yale University, New Haven, CT 06520, USA; 2 School of Natural Sciences, University of California Merced, Merced, CA, 95343, USA; 3 E. coli Genetic Stock Center, Department of Molecular, Cellular and Developmental Biology, Yale University, New Haven, CT 06520, USA; 4 Department of Microbial Pathogenesis, Yale School of Medicine, New Haven, CT 06520, USA; 5 Department of Surgery, Yale School of Medicine, New Haven, CT 06520, USA; 6 Program in Microbiology, Yale School of Medicine, New Haven, CT 06520, USA

## Abstract

Increasing prevalence and severity of multi-drug-resistant (MDR) bacterial infections has necessitated novel antibacterial strategies. Ideally, new approaches would target bacterial pathogens while exerting selection for reduced pathogenesis when these bacteria inevitably evolve resistance to therapeutic intervention. As an example of such a management strategy, we isolated a lytic bacteriophage, OMKO1, (family *Myoviridae*) of *Pseudomonas aeruginosa* that utilizes the outer membrane porin M (OprM) of the multidrug efflux systems MexAB and MexXY as a receptor-binding site. Results show that phage selection produces an evolutionary trade-off in MDR *P. aeruginosa*, whereby the evolution of bacterial resistance to phage attack changes the efflux pump mechanism, causing increased sensitivity to drugs from several antibiotic classes. Although modern phage therapy is still in its infancy, we conclude that phages, such as OMKO1, represent a new approach to phage therapy where bacteriophages exert selection for MDR bacteria to become increasingly sensitive to traditional antibiotics. This approach, using phages as targeted antibacterials, could extend the lifetime of our current antibiotics and potentially reduce the incidence of antibiotic resistant infections.

Widespread and inappropriate uses of chemical antibiotics have selected for multi-drug resistant (MDR) bacterial pathogens, presenting more frequently in human infections and contributing significantly to morbidity^1^,^2^,^3^,^4^. Some bacteria even show evolved resistance to ‘drugs of last resort’, resulting in emergent strains that are pan-drug-resistant (PDR)^5^. One example is the Gram-negative bacterium *Pseudomonas aeruginosa*, a prevalent opportunistic MDR pathogen that is poised to become a common PDR disease problem. Humans readily encounter *P. aeruginosa*, which thrives in both natural and artificial environments, varying from lakes and estuaries to hospitals and household sink drains^6^. *P. aeruginosa* causes biofilm-mediated infections, including catheter associated urinary tract infections, ventilator associated pneumonia, and infections related to mechanical heart valves, stents, grafts and sutures^7^,^8^. Individuals with cystic fibrosis, severe burns, surgical wounds and/or compromised immunity are particularly at risk for *P. aeruginosa* infections, especially acquired in hospitals^9^,^10^,^11^. *P. aeruginosa* infections are notoriously difficult to manage due to low antibiotic permeability of the outer membrane and mechanisms of antibiotic resistance that allow cross resistance to multiple classes and types of antibiotics. Arguably, the most problematic of these mechanisms is antibiotic drug efflux *via* multi-drug efflux (Mex) systems, which extrude different antibiotics that permeate the cell. Mex systems contain three components that function *via* active transport to move numerous molecules, including antibiotics, out of the cell: an antiporter that functions as a transporter (e.g., MexB, MexY), an outer membrane protein that forms a surface-exposed channel (e.g., OprM), and a periplasmic membrane fusion protein that links the two proteins (e.g., MexA, MexX)^12^. Because efflux systems such as MexAB-OprM and MexXY-OprM are able to efflux multiple classes of antibiotics^13^ and are major contributors to increased antibiotic resistance^12^,^13^,^14^,^15^,^16^, there is a pressing need to develop alternative methods for the management of antibiotic efflux of MDR *P. aeruginosa*^17^.

One alternative for treating MDR bacterial infections is phage therapy: the use of lytic (virulent) bacteriophages (bacteria-specific viruses) as self-amplifying ‘drugs’ that specifically target and kill bacteria^18^,^19^,^20^. Lytic phages bind to one or more specific receptors on the surfaces of particular bacterial hosts^18^,^20^,^21^, allowing for a targeted approach to treating bacterial infections which predated widespread use of broad-spectrum chemical antibiotics^22^. Due to the recent precipitous rise in antibiotic resistance, phage therapy has seen revitalized interest among Western physicians^23^, buoyed by successful clinical trials demonstrating safety and efficacy^21^,^24^. However, an obvious limitation to phage therapy is the abundant evidence that bacteria readily evolve resistance to phage infection^25^,^26^. While multiple mechanisms of phage resistance exist, phage attachment to a receptor binding-site exerts selection pressure for bacteria to alter or down-regulate expression of the receptor, thereby escaping phage infection^25^. Given the certainty of evolved phage-resistance, modern approaches to phage therapy must acknowledge and capitalize on this inevitability. Genetic trade-offs are often observed in biology, where organisms evolve one trait that improves fitness (a relative advantage in reproduction or survival), while simultaneously suffering reduced performance in another trait^27^,^28^,^29^. Here we propose an evolutionary-based strategy that forces a genetic trade-off: utilize phages that drive MDR bacterial pathogens to evolve increased phage resistance by suffering increased sensitivity to chemical antibiotics. Thus, this approach to phage therapy should be doubly effective; success is achieved when phage lyse the target bacterium, and success is also achieved when bacteria evolve phage resistance because they suffer increased sensitivity to antibiotics.

We predicted that phage binding to surface-exposed OprM of the MexAB and MexXY systems of MDR *P. aeruginosa* would exert selection for bacteria to evolve phage resistance, while impairing the relative effectiveness of these efflux pumps to extrude chemical antibiotics. We obtained samples from six natural sources (sewage, soil, lakes, rivers, streams, compost) and enriched for phages that could infect *P. aeruginosa* strains PA01 and PA14, two widely used MDR *P. aeruginosa* models^30^,^31^,^32^,^33^. This effort yielded 42 naturally isolated phages that successfully infected both strains of MDR *P. aeruginosa*. To test if any of these phages could bind to OprM of MexAB and MexXY efflux systems, we used a transposon knockout collection of bacterial mutants derived from *P. aeruginosa* strain PA01^34^. These assays determined which bacterial mutants failed to support phage infection, because such mutants lacked the surface-expressed protein necessary for phage infection. The assays measured the efficiency of plating (EOP), defined as the ratio of phage titer (plaque-forming units [pfu] per mL) on the knockout host relative to titer on the unaltered PA01 host. EOP ≈ 1.0 would indicate that the protein associated with the knocked out gene was irrelevant for phage binding, whereas EOP = 0 would implicate the knocked out protein as necessary for infection. Results showed that one of the 42 phage isolates failed to infect the Δ*oprM* knockout strain, but successfully infected wildtype PA01 and all other tested knockout mutants. This phage was originally isolated from a freshwater lake sample (Dodge Pond, East Lyme, Connecticut, USA). We then experimentally evolved the phage on *P. aeruginosa* strain PA01 for 20 consecutive passages, where each passage consisted of 24-hour growth on naïve (non co-evolved) bacteria grown overnight from frozen stock; this design selected for generalized improvement in phage growth but prevented possibility for host co-evolution^27^,^28^. Following serial passage, we isolated a plaque-purified sample from the evolved phage population to obtain strain OMKO1 (i.e., outer-membrane-porin M knockout dependent phage #1). We conducted whole-genome sequencing analysis of this clone and determined that phage OMKO1 had genome size ~278 kb and belonged to the dsDNA virus family *Myoviridae* (genus: *phiKZ-like-viruses*).

We next tested whether resistance to phage OMKO1 caused the desired genetic trade-off between phage resistance and antibiotic sensitivity in MDR *P. aeruginosa*. In particular, we determined whether phage resistance allowed improved killing efficiency (decreased minimum inhibitory concentration; MIC) of four antibiotics, representing four drug classes of varying capacity for efflux *via* MexAB and/or MexXY-OprM: Ceftazidime (CAZ), Ciprofloxacin (CIP), Tetracycline (TET), and Erythromycin (EM). CAZ is effluxed by the Mex system, but resistance is also inducible, determined by genetically encoded β-lactamases^35^. CIP resistance can also be regulated by multiple factors such as mutations in DNA gyrase or topoisomerase IV^36^,^37^ in addition to efflux^38^. However, resistance to TET and EM is primarily due to efflux *via* the MexAB- and MexXY-OprM efflux systems^16^,^39^. We tested effects of phage resistance on sensitivity to the four drugs in replicated assays with PA01 and PA14, as well as with three environmental strains (PAN, 1607, 1845) and three clinical isolates (PAPS, PASk, PADFU). In these assays, the phage-OMKO1 resistant strain was either a knockout mutant (∆*oprM* derived from PA01), or an independently derived spontaneous mutant of the associated parental strain.

Results for strain PA01 are shown in Fig. 1. In comparison, strain PA01 ∆*oprM* showed increased average drug sensitivity relative to PA01, in the two antibiotic environments where Mex systems provide primary (TET: 2.00 ± 0.00 μg/mL; EM: 4.667 ± 0.00 μg/mL) or moderate (CIP: 0.016 ± 0.00 μg/mL; CAZ: 0.210 ± 0.144 μg/mL) drug resistance. Thus, loss of OprM expression provided resistance to phage OMKO1, but caused greater sensitivity to all four drugs (Fold Increased Sensitivity to TET, CAZ, and EM: p < 0.01; CIP: p < 0.05) (cf. Fig. 1). The ratio of mean MIC for PA01 relative to that for ∆*oprM* was used to estimate the fold increased drug sensitivity associated with phage resistance (Fig. 1), which may be considered a baseline improvement in drug efficacy upon acquisition of phage resistance. Similar results were observed for a spontaneous phage-OMKO1 mutant of PA01 when Mex systems provided primary resistance (TET and EM; Fig. 1). As a control for transposon insertion, we examined strain ∆*mexR*, which was also derived from PA01. *mexR*, the repressor of MexAB-OprM and MexXY-OprM operons should not negatively alter phage sensitivity. As expected, this control strain was phage sensitive and our MIC assays showed inhibitory antibiotic concentrations equivalent or higher than PA01 (TET: 256.00 ± 0.00 μg/mL; EM: 256.00 ± 0.577 μg/mL; CIP: 32.00 ± 0.00 μg/mL; CAZ: 1.333 ± 0.035 μg/mL), confirming that over-expression of Mex systems improved growth in antibiotic environments where PA01 showed drug sensitivity. In addition, we examined the trade-off hypothesis in model strain PA14; for all four drugs, spontaneous phage resistance caused a statistically significant fold-increase in antibiotic sensitivity (Fig. 1). Altogether, these data showed that phage resistance led to greater drug sensitivity for antibiotics primarily controlled by Mex systems, but only sometimes improved drug efficacy when Mex systems exerted less control.

Model strains PA01 and PA14, and knockout mutants derived from these strains, are useful for elucidating mechanisms such as phage binding targets. However, microbial models inevitably experience some selection for improved fitness under controlled lab conditions, creating a potential divergence from more recently isolated clinical and environmental samples. Thus, we sought to confirm whether the desired trade-off between phage-OMKO1 resistance and increased drug sensitivity occurred in environmental and clinical strains. After determining that the clinical and environmental strains were sensitive to phage OMKO1, we isolated spontaneous phage-resistant mutants of each strain, and conducted MIC assays. Results (Fig. 1) confirmed that resistance to phage OMKO1 coincided with increased sensitivity of each environmental isolate to antibiotics TET and EM. Phage resistance led to greater drug sensitivity for two clinically relevant antibiotics (CAZ, CIP), with the majority of outcomes showing statistical significance (Fig. 1). Importantly, the phage resistant mutants of all three of the clinical isolates (PAPS, PASk, PADFU) showed significantly increased drug sensitivity to the tested antibiotics. Thus, results for the environmental and clinical isolates qualitatively matched those observed in the well-characterized strains PA01 and PA14, suggesting that phage OMKO1 is generally capable of forcing the desired genetic trade-off in MDR *P. aeruginosa*.

We further compared the effects of phage sensitivity versus resistance on *P. aeruginosa* fitness, by examining growth kinetics of bacterial mutants in antibiotic medium when phage OMKO1 was either present or absent. We obtained bacterial growth curves by monitoring changes in optical density (OD_600_) in liquid culture. These assays challenged knockout strains ∆*mexR* and ∆*oprM* to grow in a TET (10 μg/mL) environment, where phage OMKO1 was either present or absent. Results (Fig. 2) confirmed that over-expression of Mex systems allowed robust growth of populations founded by strain ∆*mexR* in the presence of TET. However, as expected these phage sensitive populations grew three-fold worse and highly similar to the ∆*oprM* population in an identical drug environment containing phage OMKO1 (Fig. 2). Phage presence did not completely eliminate ∆*mexR* bacteria, perhaps explained by persistence of spontaneous phage resistant mutants that suffered the desired trade-off and failed to increase in density during the assay. Phage resistant populations founded by strain ∆*oprM* showed impaired growth in the TET environment due to the knocked out OprM component of the Mex system. As expected, presence of phage OMKO1 had no effect on growth kinetics of ∆*oprM* populations, because the virus was incapable of binding to these cells. In both cases, the observed weak growth of ∆*oprM* populations in TET environments was perhaps due to the low permeability of *P. aeruginosa* cell membranes, which is problematic for treatment of these infections using antibiotics alone.

To evaluate whether phage OMKO1 would be broadly useful in targeting *P. aeruginosa* strains we examined the conservation of the MexAB- and MexXY-OprM efflux systems. To do so, we estimated effects of selection on five genes encoded in these Mex systems (*oprM*, *mexA*, *mexB*, *mexX*, *mexY*) using genetic data from 38 *P. aeruginosa* strains representing the known genetic diversity of *P. aeruginosa* queried from NCBI GenBank. For each gene, this analysis measured ω (d_N_/d_S_): the ratio of the number of non-synonymous substitutions per non-synonymous site (d_N_) to the number of synonymous substitutions per synonymous site (d_S_), which is used to indicate selective pressure acting on a protein-coding gene. Results (Table 1) showed that strong stabilizing selection was acting on *oprM*, *mexA*, *mexB*, and *mexX* genes, such that none of these loci were observed to be changing under positive selection. These data indicated that the structure of the OprM protein was strongly constrained to remain stable through time; thus, phage OMKO1 should be capable of infecting a wide variety of *P. aeruginosa* genotypes due to genetic stability of the binding target. Furthermore, the analysis suggested low probability of wildtype functionality for novel mutations that would confer *P. aeruginosa* resistance to phage OMKO1 *via* alteration of the OprM attachment site. Nevertheless, it is plausible that even highly conserved genes in bacteria can transiently change in response to intermittent selection pressures (such as phages), while leaving no signs of rapid evolution within the gene sequence. For example, a gene may acquire a short duplicated region that interrupts its function, and in *oprM* such a mutation could simultaneously cause efflux pump deficiency while conferring phage resistance. Because small duplications within a gene may revert rapidly, this dynamic process could allow efficient restoration of the original phenotype, fostering bacterial ability to phenotypically change in response to prevailing selection pressure while leaving no signs of rapid evolution within the gene sequence. This example is hypothetical, and future studies will be necessary to elucidate precise mechanisms by which *P. aeruginosa* may evade the trade-off observed in our study. Regarding the other genes in our analysis, we noted that strong positive selection was detected only for *P. aeruginosa* gene *mexY*, for unknown reasons but indicating that this component of MexXY is changing relatively rapidly.

Our study showed that phage OMKO1 is a naturally occurring virus that forces a desired genetic trade-off between phage resistance and antibiotic sensitivity, which should benefit phage therapy efforts against MDR bacteria such as *P. aeruginosa*. Isolation of phage OMKO1 from nature suggested that other phages might have evolved to utilize OprM or other surface-exposed proteins of Mex systems as binding sites. These types of phage could be highly useful for developing therapeutics, because target bacteria are expected to inevitably evolve phage resistance resulting in antibiotic susceptibility. Previous studies similarly demonstrated the evolutionary interplay between phage selection and maintenance of antibiotic resistance in bacterial pathogens. For example, phage binding may rely on surface proteins coded by plasmid genes, causing phage to select against plasmid maintenance in bacterial populations, thereby reducing the prevalence and spread of plasmid-borne antibiotic resistance genes^40^. Other studies also suggest that combined use of phages and antibiotics is superior to either selection pressure alone, indicating that the dual approach is promising as an antimicrobial strategy^41^,^42^. Our study demonstrates that phage OMKO1 is also a promising evolutionary-based phage adjunctive, which can be used to directly exploit a genetic trade-off between efflux mediated antibiotic resistance and phage resistance. Taken together, these examples illustrate the potentially valuable approach by which an evolutionary-based antibiotic adjunctive could greatly improve clinical outcomes and reduce the spread of antibiotic resistant infections. The clinical utility of phages such as OMKO1 is vital because selection using this phage restores usefulness of antibiotics that are no longer considered to be therapeutically valuable. In the past, there was an attempt to restore waning amoxicillin efficacy, by combining this drug with clavulanic acid (a β-lactamase inhibitor). Although clavulanic acid has minimal antibacterial activity, it interacts with β-lactamase enzyme *via* mechanism-based inhibition, allowing amoxicillin to inhibit cell wall synthesis. While this therapeutic approach often can be effective as demonstrated by more than 30 years of successful use of amoxicillin/clavulanic acid, the negligible antibacterial activity of clavulanic acid exerts selection pressure for hyper-production of β-lactamase as the means for bacteria to successfully evolve resistance to the adverse effects of clavulanic acid^43^. In contrast, a phage therapy approach exerts selection pressure in the desired direction, causing bacteria to become increasingly antibiotic sensitive and allowing for renewed use of historically effective antibiotics that have been rendered useless by the evolution of antibiotic resistance. Furthermore, our approach suggests that antibiotics not typically used during treatment of *P. aeruginosa* infections due to intrinsic resistance^44^ could be used with phage OMKO1. This method effectively ‘re-discovers’ a class of antibiotics that has already been clinically tested/approved. Consequently, this approach has the potential to extend the effective lifetime of antibiotics in our ‘drug arsenal’ and broaden the spectrum of these drugs, greatly reducing the burden on drugs of last resort, preserving them for future use. Ideally, phage therapy that utilizes phages such as OMKO1 would not only improve clinical efficacy against MDR bacteria, but also could potentially slow or reverse the incidence of antibiotic resistant bacterial pathogens.

## Materials and Methods

### *Pseudomonas aeruginosa* strains

*P. aeruginosa* strains PA01 and PA14 were kindly provided by B. Kazmierczak (Yale School of Medicine). Strains derived from PA01 that each contained a knockout of a gene in the Mex system were obtained from the *Pseudomonas aeruginosa* PA01 Transposon Mutant Library (Manoil Lab, University of Washington).

*P. aeruginosa* PAPS was collected from fistular discharge of a patient with a history of chronic infection associated with an aortic arch replacement surgery. This strain was associated with a biofilm that formed on an indwelling Dacron aortic arch and has been present for >1 year in the patient. *P. aeruginosa* PASk was collected from an open wound on the skull of a 60 y.o. male that was not responsive to antibiotic therapy or hyperbaric oxygen. *P. aeruginosa* PADFU was collected from a diabetic foot ulcer. These strains were collected from consented donors and de-identified. Furthermore, experiments were performed in accordance with The Yale University Human Investigation Committee/Institutional Review Board (HIC/IRB) guidelines, and relevant experimental protocols were approved by Yale’s HIC/IRB committee.

*P. aeruginosa* strains 1845 and 1607 were collected from household sink drains (1845: bathroom sink drain; 1607: Kitchen sink drain) in a previous study^6^, and kindly provided by S. Remold, University of Louisville.

### Challenge assays using knockout library of *P. aeruginosa*

The transposon knockout mutants used for screening included 11 strains, which differed in the knockout of a gene for a surface expressed protein in the Mex system: *oprC*, *oprB*, *oprG*, *oprD*, *oprI*, *oprH*, *oprP*, *oprO*, *oprM*, *oprJ*, *oprN*. Also, we tested phage ability to grow on 8 strains that differed in the knockout of a gene for an internal protein of the Mex system: *mexH*, *mexA*, *mexB*, *mexR*, *mexC*, *mexD*, *mexE*, *mexF*. These replicated (*n* = 3) assays calculated the average efficiency of plating (EOP) on a knockout host: plating ability (titer in plaque-forming units *per* mL) for a phage on the test knockout strain, relative to its plating ability on a phage sensitive host (PA01).

### Isolation of phage OMKO1

The phage isolated from Dodge Pond was serially passaged on host strain PA01 for 20 consecutive passages. To do so, PA01 was grown to exponential phase in 25 ml of Luria-Bertani (LB) broth and then infected with phage at multiplicity of infection (MOI; ratio of phage particles to bacterial cells) of ~0.1, using 37 °C shaking (100 rpm) incubation. After 12 hours, the culture was centrifuged and filtered (pore size: 0.22 μm) to remove bacteria, and to obtain a cell-free lysate. The next passage was initiated under identical conditions, using naïve (non-coevolving) PA01 bacteria grown fresh from frozen stock. This process was continued for 20 passages total, and phage OMKO1 was plaque purified from the endpoint phage population.

### Isolation of phage resistant mutants

Phage OMKO1 was amplified on *P. aeruginosa* in liquid culture in conditions identical to the *Serial passage* assays. Following 12 hours of amplification, 100 μl of culture was plated on LB agar and incubated for 12 hours. Individual colony-forming units (CFUs) were then collected, and verified to be phage resistant by classic ‘spot tests’ (i.e., 10^7^ PFU of phage OMKO1 was pipetted onto a lawn of each bacterial isolate to test whether the phage was capable of visibly clearing the lawn [indicating bacterial sensitivity to phage] versus incapable of clearing the lawn [indicating bacterial resistance to phage]).

### Minimum inhibitory concentration assays

Bacterial strains were grown overnight at 37 °C as described above. A 200 μL sample of the culture was then spread onto an LB agar plate, and allowed to dry for 10 minutes, followed by application of an eTestStrip (BioMerieux) for a test antibiotic. Plates were incubated at 37 °C for 12 hours, and MIC was estimated as the point at which bacterial growth intersected the eTest strip. Each strain was tested in triplicate for each antibiotic.

### Bacterial growth kinetics

Bacterial growth was assayed using a TECAN Freedom EVO workstation (TECAN Schweiz AG, Männedorf, Switzerland), which included an automated spectrophotometer (TECAN INFINITE F200 plate-reader) to monitor changes in bacterial density (optical density = OD_600_) and a Robotic Manipulator Arm (RoMA) to manipulate cultures grown in 96-well flat-bottomed optical plates (Falcon). Each test strain was grown in LB broth with replication (*n* = 3), and some assays included bacteria mixed with phage OMKO1 at an MOI ~10 to increase the probability that all susceptible bacteria in the well were initially infected. Assays were controlled via scripts prepared in TECAN’s Freedom EVOWare and iControl software. Plate incubation occurred at 37 °C with 5 Hz continuous shaking in incubation ‘towers’. Every 2 min, each plate was sequentially transferred by the RoMA to the plate reader to measure OD. Within the plate reader, prior to OD reading the plate was shaken orbitally at 280 rpm and with 2 mm amplitude for 10 seconds. Absorbance wavelength was measured at 620 nm over the course of 15 flashes, and the resulting OD for each well was outputted by iControl into a time-stamped delimited text file, which was then imported to Excel (Microsoft) for further analysis. The plate was then transferred by RoMA back to the incubation tower, and the protocol was repeated for 12 hours total.

### Bioinformatics analysis

Syntenic copies of the genes *oprM*, *mexA*, *mexB*, *mexX* and *mexY* were extracted from 38 publicly available genomes (Appendix 1) of *P. aeruginosa*, representing a cross section of the extant genetic diversity of the species. These sequences were aligned using MUSCLEv3.8.31^45^ and refined by eye. Maximum likelihood trees were estimated for each gene using RaxMLv8.0.0^46^. The *d*_*N*_*/d*_*S*_ (*ω*) ratio for each gene was calculated using the codeML of PAMLv4.8^47^ using model M2a with *ω* both fixed and variable. Significance of positive selection for each gene was evaluated by conducting a likelihood ratio test of the likelihood values implemented in the base package of R software v. 3.2.1.

## Additional Information

**How to cite this article**: Chan, B. K. *et al*. Phage selection restores antibiotic sensitivity in MDR *Pseudomonas aeruginosa*. *Sci. Rep*. **6**, 26717; doi: 10.1038/srep26717 (2016).

## Supplementary Material

Supplementary Information

## Figures and Tables

**Figure 1:**
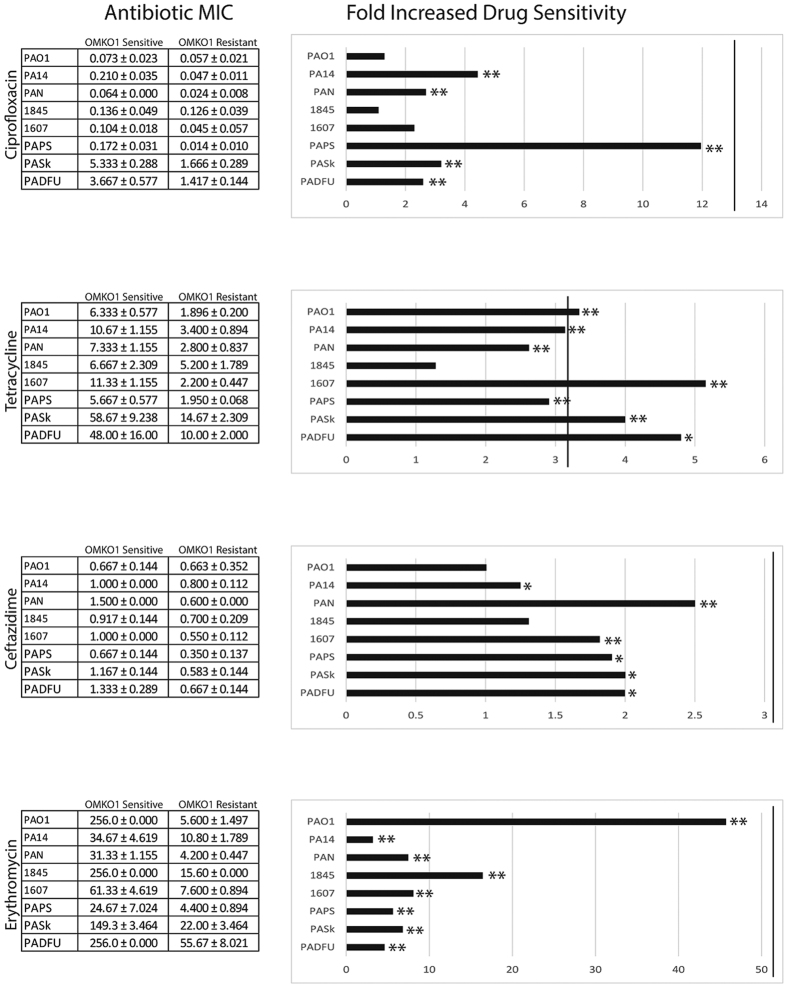
Selection for phage resistance causes a trade-off resulting in significantly reduced Minimum Inhibitory Concentrations (MIC) to four drugs drawn from different antibiotic classes. LEFT: Average MIC ± SD of four antibiotics for phage sensitive MDR bacteria (left column) and for spontaneous mutants of these bacteria resistant to phage OMKO1 (right column). RIGHT: Fold improvement of MIC for isolated strains resistant to OMKO1 (*p < 0.05, **p < 0.01). For comparison, data for fold-increased sensitivity of transposon knockout PAO1-∆*oprM* (phage resistant) is displayed as a vertical black line.

**Figure 2:**
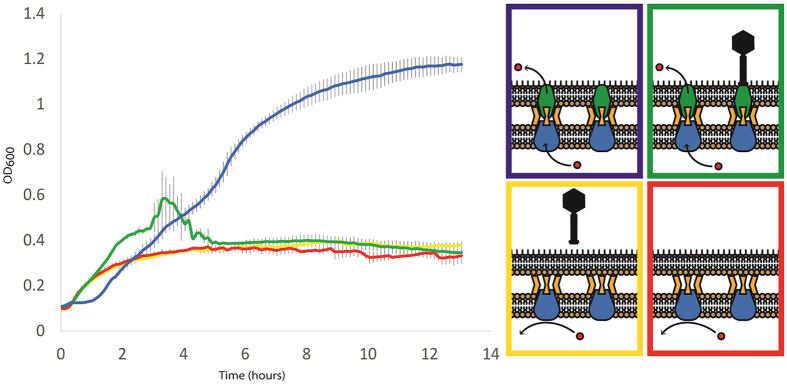
Phage OMKO1 selects against the expression of OprM and, consequently, the function of the mexAB/XY-OprM efflux systems. Average cell densities (OD_600_) of PA01-Δ*mexR* and PA01-Δ*oprM* over time in the presence of Tetracycline (10 mg/L) and phage OMKO1 (green and red lines). PAO1 ∆*mexR* (blue, green) overexpresses mex-OprM and readily grows in TET to high densities alone due to active efflux of TET (blue) but is susceptible to phage infection (green). PAO1 ∆*oprM* grows poorly in the presence of TET (red) but is resistant to phage OMKO1 (yellow).

**Table 1:** Evaluation of selection acting upon genes associated with MexXY- and MexAB-OprM efflux systems of *P. aeruginosa*.

Gene		*p*_*0*_	*p*_*1*_	*p*_*2*_ = 1 − *p*_*0*_ − *p*_*1*_	ΔLRT	p
*oprM*	*p*	0.996	0.001	0.003	0.000	1.000
	ω	0.010	1.000	1.000		
*mexA*	*p*	1.000	0.000	0.000	0.000	1.000
	ω	0.005	1.000	1.000		
*mexB*	*p*	0.989	0.000	0.011	2.677	0.102
	ω	0.004	1.000	1.000		
*mexX*	*p*	0.971	0.014	0.015	3.631	0.057
	ω	0.023	1.000	1.000		
*mexY*	*p*	0.316	0.636	0.048	170.230	<0.001
	ω	0.000	1.000	17.849		

A bioinformatics approach tested whether data from published *P. aeruginosa* genomes showed conservation among *oprM*, *mexAB* and *mexXY* genes. *p*: proportion of variable sites assigned to each class in the selection model, *ω*: the overall *d*_*N*_*/d*_*S*_ ratio for the variable sites which fit into each site class for each alignment. Selection models P_0_: purifying selection, P_1_: neutral selection: P_2_: positive selection. ΔLRT: the difference in likelihood ratios between fixed and estimated *ω* values and associated p value for the presence of positive selection.
