# Supplementary material for: Phage selection restores antibiotic sensitivity in MDR Pseudomonas aeruginosa
Source: Sci Rep. 2016 May 26;6:26717. doi: 10.1038/srep26717 (PMC4880932; doi:10.1038/srep26717)
Supplement: Supplementary Information [file srep26717-s1.pdf]

Title: Phage selection restores antibiotic sensitivity in MDR *Pseudomonas aeruginosa*

Authors: Benjamin K. Chan[1], Mark Sistrom[2], John E. Wertz[3], Kaitlyn E. Kortright[4], Deepak Narayan[5], and Paul E. Turner[1,6]*

Supplementary Information

Appendix 1: Summary of strains used for the selection analysis, including GenBank Assembly number, source and country, date and isolation notes where known.

Strain Name	**GenBank Accession**	**Source**	**Country**	**Collection Date**	**Notes**
VRFPA01	GCA_000335395.3	Sankara Nethralaya Vision Research Foundation	India	2012	blood isolate from Indian septicemia patient
AZPAE15042	GCA_000790465.1	AstraZeneca	USA	unknown	cystic fibrosis isolate
PA7	GCA_000017205.1	J. Craig Venter Institute	Argentina	unknown	non-respiratory clinical isolate
19660	GCA_000481765.1	Broad Institute	USA	unknown	Cornea/ocular infection
19br	GCA_000223945.2	IBIS, Universite Laval	unknown	unknown	unknown
AZPAE14903	GCA_000791145.1	AstraZeneca	Spain	2008	itra-abdominal tract infection
P2-L230/95	GCA_000760505.2	Center for Cellular and Molecular Biology(CCMB)	India	1995	Obtained from Keratitis Patient
PA38182	GCA_000531435.1	University of London	UK	unknown	resistant to antibiotics other than colistin
X24509	GCA_000481865.1	Broad Institute	USA	unknown	UTI patient
BWHPSA028	GCA_000481145.1	Broad Institute	USA	2013	Isolated from Sputum
C41	GCA_000480455.1	Broad Institute	unknown	unknown	Environmental isolate
CF27	GCA_000481905.1	Broad Institute	USA	unknown	Cystic fibrosis patient
BWHPSA022	GCA_000481265.1	Broad Institute	USA	2013	Sputum isolated
DQ8	GCA_000283055.1	Shanghai Jiao Tong University	China	unknown	soil isolated
PA01	GCA_000006765.1	PathoGenesis Corporation	Australia	1955	wound isolated
PDR	GCA_000783275.1	China CDC	China	unknown	isolate from a patient with urinary infection
BWHPSA037	GCA_000520455.1	Broad Institute	USA	2013	bronchoalveolar lavage
CF77	GCA_000480375.1	Broad Institute	USA	2005	cystic fibrosis isolate
LESB58	GCA_000026645.1	Wellcome Trust Sanger Institute	UK	1988	cystic fibrosis isolate
NCMG1179	GCA_000291745.1	National Center for Global Health and Medicine	Japan	2010	isolated from inpatient respiratory tract
AZPAE14698	GCA_000794705.1	AstraZeneca	Israel	unknown	respiratory tract infection

C23	GCA_000480495.1	Broad Institute	unknown	unknown	Environmental isolate
PS42	GCA_000520195.1	Broad Institute	Venezuela	unknown	Environmental isolate
Stone130	GCA_000478465.2	Broad Institute	unknown	unknown	unknown
B13633	GCA_000359505.1	National Tsing Hua University	unknown	unknown	infant with community-acquired diarrhea
VRFPA04	GCA_000473745.3	Vision Research Foundation, Sankara Nethralaya	India	unknown	Isolated from Human corneal button
BL04	GCA_000481065.1	Broad Institute	USA	unknown	isolated from eye
PABL056	GCA_000290555.1	Northwestern University Feinberg School of Medicine	USA	2001	isolated from blood
BL13	GCA_000480885.1	Broad Institute	USA	unknown	isolated from eye
P7L63396	GCA_000760495.2	Center for Cellular and Molecular Biology(CCMB)	India	1996	isolate from keratitis patient
VRFPA03	GCA_000467675.1	Vision Research Foundation, Sankara Nethralaya	India	2012	Corneal button from corneal keratitis patient
VRFPA09	GCA_000558345.1	Vision Research Foundation, Sankara Nethralaya	India	2013	blood sample from patient with septicaemia
BL03	GCA_000481085.1	Broad Institute	USA	unknown	Corneal Scaping
39016	GCA_000148745.1	Centre for Genomics Research, University of Liverpool	unknown	unknown	cornea of a patient with ulcerative keratitis
AZPAE13850	GCA_000795435.1	AstraZeneca	India	unknown	unknown
BL25	GCA_000480645.1	Broad Institute	USA	unknown	isolated from eye
AZPAE14699	GCA_000794725.1	AstraZeneca	USA	2012	itra-abdominal tract infection
UCBPPPA14	GCA_000014625.1	Massachusetts General Hospital	USA	unknown	Human clinical isolate
